# Interdependent Impact of Lipoprotein Receptors and Lipid-Lowering Drugs on HCV Infectivity

**DOI:** 10.3390/cells10071626

**Published:** 2021-06-29

**Authors:** Francisco J. Zapatero-Belinchón, Rina Ötjengerdes, Julie Sheldon, Benjamin Schulte, Belén Carriquí-Madroñal, Graham Brogden, Laura M. Arroyo-Fernández, Florian W. R. Vondran, Benjamin Maasoumy, Thomas von Hahn, Gisa Gerold

**Affiliations:** 1 Centre for Experimental and Clinical Infection Research, Institute for Experimental Virology, TWINCORE, 30625 Hannover, Germany; Francisco.Zapatero@tiho-hannover.de (F.J.Z.-B.); Rina.M.Oetjengerdes@stud.mh-hannover.de (R.Ö.); julie.sheldon@twincore.de (J.S.); Belen.Carriqui@tiho-hannover.de (B.C.-M.); Graham.Brogden@tiho-hannover.de (G.B.); 2 Department of Gastroenterology, Hepatology and Endocrinology, Hannover Medical School, 30625 Hannover, Germany; Schulte.Benjamin@mh-hannover.de (B.S.); Maasoumy.Benjamin@mh-hannover.de (B.M.); 3 Institute of Molecular Biology, Hannover Medical School, 30625 Hannover, Germany; 4 German Center for Infection Research (DZIF), Hannover-Braunschweig Site, 38124 Braunschweig, Germany; Vondran.Florian@mh-hannover.de; 5 Department of Clinical Microbiology, Virology, Umeå University, SE-90185 Umeå, Sweden; 6 Wallenberg Centre for Molecular Medicine (WCMM), Umeå University, SE-90185 Umeå, Sweden; 7 Department of Biochemistry & Research Center for Emerging Infections and Zoonoses (RIZ), University of Veterinary Medicine Hannover, 30559 Hannover, Germany; Laura.Maria.Arroyo.Fernandez@tiho-hannover.de; 8 Department of General, Visceral and Transplant Surgery, Hannover Medical School, 30625 Hannover, Germany; 9 Department of Gastroenterology, Hepatology and Interventional Endoscopy, Asklepios Hospital Barmbek, Semmelweis University, Campus Hamburg, 22307 Hamburg, Germany

**Keywords:** hepatitis C virus, SR-B1, LDLr, lipid metabolism, lipoprotein receptor, lipid-lowering drug, statin, fibrate, PCSK9-inhibitor, HMG-CoA-reductase inhibitor, HCV

## Abstract

The HCV replication cycle is tightly associated with host lipid metabolism: Lipoprotein receptors SR-B1 and LDLr promote entry of HCV, replication is associated with the formation of lipid-rich membranous organelles and infectious particle assembly highjacks the very-low-density lipoprotein (VLDL) secretory pathway. Hence, medications that interfere with the lipid metabolism of the cell, such as statins, may affect HCV infection. Here, we study the interplay between lipoprotein receptors, lipid homeostasis, and HCV infection by genetic and pharmacological interventions. We found that individual ablation of the lipoprotein receptors SR-B1 and LDLr did not drastically affect HCV entry, replication, or infection, but double lipoprotein receptor knock-outs significantly reduced HCV infection. Furthermore, we could show that this effect was neither due to altered expression of additional HCV entry factors nor caused by changes in cellular cholesterol content. Strikingly, whereas lipid-lowering drugs such as simvastatin or fenofibrate did not affect HCV entry or infection of immortalized hepatoma cells expressing SR-B1 and/or LDLr or primary human hepatocytes, ablation of these receptors rendered cells more susceptible to these drugs. Finally, we observed no significant differences between statin users and control groups with regards to HCV viral load in a cohort of HCV infected patients before and during HCV antiviral treatment. Interestingly, statin treatment, which blocks the mevalonate pathway leading to decreased cholesterol levels, was associated with mild but appreciable lower levels of liver damage markers before HCV therapy. Overall, our findings confirm the role of lipid homeostasis in HCV infection and highlight the importance of the mevalonate pathway in the HCV replication cycle.

## 1. Introduction

Hepatitis C virus (HCV) infects 71 million people and causes approximately four hundred thousand deaths annually [1]. Chronic infection can lead to hepatitis, liver cirrhosis, and hepatocellular carcinoma, and is the leading cause of liver transplantation worldwide [2]. Since the discovery and approval of direct-acting antiviral agents (DAA) [3], almost all infections can be cured by drug treatment if available [4,5]. Nonetheless, to this day, HCV remains a highly prevalent pathogen that is uniquely adapted to its human host and able to evade the host immune system without causing general immunosuppression.

The ability of HCV to fly under the radar of the immune system may partly be due to the HCV replication cycle being tightly intertwined with the host’s lipid metabolism. In HCV-infected patients, HCV is found in association with very-low-density lipoprotein (VLDL) and low-density lipoprotein (LDL) in the so-called lipoviral particle (LVP) [6,7], which may shield virions from immune recognition. Inside the cell, HCV replication is associated with the formation of a double-membrane replication compartment known as “membranous web” [8]. During HCV assembly, viral structural and non-structural proteins highjack lipid droplets and the VLDL secretory pathway for the assembly and secretion of the LVPs [9,10].

Interestingly, these HCV LVPs have been proposed to exploit LDL and high-density lipoprotein (HDL) receptors, namely scavenger receptor class B type 1 (SR-B1) and low density lipoprotein receptor (LDLr), for productive cell entry [11,12,13]. In agreement with this model, both LVP-embedded apolipoprotein E (apoE) and viral envelope glycoprotein E2 have been shown to bind to SR-B1 and thereby facilitate subsequent binding of E2 to CD81 [11,14]. On the other hand, the role of LDLr in the HCV entry life cycle is still controversial [15,16,17]. Whereas some studies have proposed that LDLr mediates cell attachment via the LVP component apoE [16,17], others have reported that LDLr may have a role in viral replication [15]. Finally, a landmark study by Yamamoto and colleagues indicated, for the first time, that LDLr and SR-B1 may have a redundant role in HCV entry [13]. In this study, generation of individual genetic knock-outs (KOs) of LDLr or SR-B1 had a modest impact on HCV infection, but genetic ablation of both receptors had a much more pronounced effect. Importantly, this effect could be rescued by overexpression of either SR-B1 or LDLr individually. However, the effect of genetic ablation of lipoprotein receptors on cellular cholesterol content and expression of HCV entry factors remained elusive.

Lipid-lowering drugs (LLDs) are used for the treatment of hyperlipidemia with LDL being the most important target lipid species since its levels are strongly associated with cardiovascular risk [18]. Since the liver plays a central role in lipid metabolism, LLDs impact liver homeostasis (including lipoprotein receptor expression) and consequently, they may influence permissiveness of hepatocytes to HCV infection. In addition, altered serum lipoprotein levels may influence the infectivity of HCV LVPs. Currently, there are mainly three different classes of LLD in clinical use: statins, fibrates, and proprotein convertase subtilisin/kexin type 9 (PCSK9) inhibitors [18]. These LLDs decrease blood LDL levels by different mechanisms. Statins are known to interact with the binding site of 3-hydroxy-3methyl-glutaryl-coenzyme A (HMG-CoA) reductase and thereby inhibit the mevalonate pathway which leads to a decreased de novo cholesterol synthesis in hepatocytes and consequently increased expression of cell surface LDLr [19,20]. Fibrates induce the activation of the peroxisome proliferator-activated receptor alpha (PPAR-α) transcription factors which in turn activate the synthesis of HDL and increase the activity of lipoprotein lipases [21]. Furthermore, fibrates increase fatty acid uptake, reduce triglyceride production, decrease exchange of cholesteryl ester and triglyceride between VLDL and HDL, and increase the affinity between LDLr and LDL [22]. Lastly, PCSK9 is an enzyme that promotes LDLr cell surface internalization and lysosomal degradation [23,24]. PCSK9-inhibitors are humanized antibodies (abs) that specifically bind and block serum PCSK9 resulting in a higher density of LDLr on the cell surface [25].

The effects of individual LLDs on the HCV replication cycle have been previously addressed [26,27,28,29,30,31,32,33]. Statins seem to have a multimodal mode of action with regard to HCV replication [26,27,28]. For instance, it has been shown that inhibition of geranylgeranylation by statins hinders HCV replication [26]. Alternatively, Blanchet et al. described a dual proviral/antiviral effect on the entry of HCV that was dependent on the drug dose in vitro. At low concentrations statins upregulate LDLr and thereby HCV entry, while at higher concentrations statins downregulate the HCV entry cofactor Claudin-1 (CLDN1) and inhibit virus entry [27]. Finally, Wuestenberg and colleagues showed that statins inhibit HCV replication in vitro through the induction of the heme degrading enzyme heme oxygenase 1 (HO-1) and induction of the interferon response [28]. The combination of statins with interferon or DAAs has synergistic effects that reduce HCV RNA levels and prevent the rise of resistant variants [29,30]. Interestingly, all these in vitro studies observed a lack of antiviral activity for pravastatin suggesting that the suppression of HMG-CoA reductase activity might not be the main determinant of HCV inhibition [26,27,28]. 

In vivo, the efficiency of statins is strongly debated [34,35,36]. A retrospective study of HCV chronically infected patients routinely attending the clinic failed to show a correlation between statin therapy and decreasing viral loads [35]. However, in a randomized controlled trial, combination of pegylated interferon therapy with statins increased the sustained viral response (SVR) and reduced the relapse rate of chronically infected patients [37]. Moreover, an analysis of the longitudinal Electronically Retrieved Cohort of HCV Infected Veterans (ERCHIVES) revealed that patients treated with statins in addition to HCV therapy had a higher SVR and lower liver disease progression over 24 months after HCV treatment [36]. Although the interplay between statins and HCV has been extensively studied, the impact of fibrates and PCSK9 inhibitors during HCV infection is much less clear. For fibrates, early reports suggested that fenofibrate intervention increases secretion of apolipoprotein all (apoAll) and HCV core (C) in HepG2 cells [32]. It was later shown that clofibrate intervention of humanized C/OTg mice inhibit HCV replication by activation of antiviral host factor quinolinate phosphoribosyl transferase (QPRT) [33]. Although initial clinical studies suggested a beneficial effect of bezafibrate addition on serum viral loads of HCV infected patients, it was subsequently shown that this observation was associated with lower liver injury levels and higher liver functionality [38,39]. Lastly, the only study dealing with the fully humanized α-hPCSK9 monoclonal ab (mab) alirocumab suggested that this mab does not affect HCV entry and replication, refuting previous reports that suggested a reduction of CD81 cell surface expression and HCV infectivity after ectopic expression of a membrane-bound version of PCSK9 [31,40]. Taken together, since LLDs are among the most commonly used drugs worldwide, it is imperative to understand the interplay between LLDs, hepatocyte lipid homeostasis and HCV infectivity.

In this study, we aimed to understand the effect of different classes of LLDs at clinically relevant concentrations and under different genetic conditions on HCV infection in vitro and in vivo. Statin simvastatin, fibrate fenofibrate, and PCSK9 inhibitor alirocumab had marginal or no effect on HCV glycoprotein (GP)-mediated entry or cell-culture derived HCV (HCVcc) infection of hepatoma cells (Huh-7.5) or primary human hepatocytes (PHH). Whereas individual genetic ablation of either LDLr or SR-B1 did not affect HCV infectivity drastically, a double lipoprotein receptor KO (DKO) notably decreased HCV infection. We could show that this effect was not due to alterations of HCV cell entry co-factor expression or cellular cholesterol content. Treatment of these lipoprotein receptor-deficient cells with simvastatin and fenofibrate led to an additive reduction of HCV infectivity, suggesting that cholesterol level alterations or lipid homeostasis caused the observed HCV inhibition. Lastly, retrospective analysis of a cohort of patients treated with DAA-based HCV therapy failed to show a correlation between HCV RNA levels and statin treatment but suggests a modest beneficial effect of statins on liver integrity before DAA therapy. In summary, our results underline the importance of lipid homeostasis for HCV infection and suggest a link between de novo cholesterol synthesis and HCV infection.

## 2. Materials and Methods

### 2.1. Cell Lines

Huh-7.5 and Huh-7.5.1 adherent hepatoma cell lines were kindly donated by Charles M. Rice (Rockefeller University, New York City, NY, USA). Parental Huh-7.5 derived lipoprotein receptor KOs SR-B1 #1, #2, #3, #4; LDLr KO #1, #2, #3, #4, and DKO #1, #2, #3 cell lines were cultivated in Dulbecco’s modified eagle medium (DMEM) (Gibco, Gaithersburg, MD, USA) supplemented with 10% Fetal Calf Serum (FCS) (Sigma–Aldrich, St. Luis, MO, USA; Capricorn Scientific, Ebsdorfergrund, Germany), non-essential amino acids, penicillin/streptomycin (100 µg/mL) and L-Glutamine (2 mM) (Gibco, Gaithersburg, MD, USA) at 37 °C and 5% CO_2_. From here on this media formulation will be denominated DMEM complete.

### 2.2. DNA Plasmids

HIV-based gutted Gag-Pol packaging construct, transfer plasmids CSPW (Puromycin resistance gene Puro^r^) and pWPI_NanoLuc_BLR (NanoLuciferase), VSV-G, MLV env, HCV J6 E1-E2, or HCV H77 E1-E2 glycoprotein expressing vector, and pCDNA3.1 empty backbone have been previously described [41,42,43]. Full-length Jc1 firefly luciferase (FLuc) reporter construct pFKi389LucEiJFH1/J6/C-846 and HCV Con1 (pFKi 389 Neo EI Core 3′JFH1wt) have been previously described [44,45]. HCV replicon constructs pFK-Luc-JFH1/ΔE1-E2 and pFK-Luc-JFH1/ΔE1-E2 ΔGDD were previously described [45]. The CRISPR kit used for constructing multiplex CRISPR/Cas9 single and double knock-out (KO) vectors was a gift from Takashi Yamamoto (Addgene kit # 1000000055) [46]. Target sequence oligonucleotides TTTGGAGTCAACCCAGTAG and TCATGAAGGCACGTTCGCCG against *LDLR* exon 12 and *SCARB1* exon 4 were respectively cloned by golden gate assembly into pX330A-1x2 and pX330S-2 according to the author’s protocol [46]. Briefly, 10 µM annealed oligonucleotides were mixed with 25 ng/µL vector, BbsI restriction enzyme (NEB, Ipswich, Massachusetts, USA), and T4 ligase buffer and enzyme (Thermo Fischer Scientifc, Waltham, Massachusetts, USA). Reaction was run for 3 cycles at 37 °C for 5 min and 16 °C for 10 min. Resulting products were transformed in DH5α E. coli bacteria and clones were Sanger sequenced for correct nucleotide sequence. For the generation of *LDLR*/*SCARB1* double KO vector pX330A-1x2/LDLRgRNA3-SCARB1gRNA2, 50 ng/µL of pX330A-1x2/LDLRgRNA3 and 100 ng/µL of pX330S-2/SCARB1gRNA2 were mixed with BsaI restriction enzyme (NEB, Ipswich, MA, USA), T4 ligase buffer and enzyme and run at 37 °C for 5 min, 16 °C for 10 min for 25 cycles. Product was transformed and sequenced verified.

### 2.3. Generation of Lipoprotein Receptor-Deficient Cell Lines

To generate LDLr, SR-B1 and DKO Huh-7.5, cells were seeded into a 6-well format at 8 × 10^5^ cells/well confluency. The next day, pX330A-1x2/LDLRgRNA3, pX330S-2/SCARB1gRNA2, and pX330A-1x2/LDLRgRNA3-SCARB1gRNA2 constructs were individually co-transfected with Puro^r^ expressing vector CSPW at a 3:1 ratio with FUGENE^®^ HD reagent (Promega, Madison, WI, USA) (FUGENE 5:1 DNA) according to the manufacture’s protocol. 12 h post-transfection, media was removed and fresh DMEM complete media supplemented with 4 µg/mL Puromycin was added to the transfected and non-transfected control cells. 48 h post-treatment (time after all non-transfected control cells were dead), selection media was removed and cells were left to recover for 72 h. Subsequently, serial dilution-based single cell cloning was performed on each treatment. Individual clones were expanded and KO generation verified by cell surface staining and immunoblotting of the target proteins.

### 2.4. HCV Virus Production and Infection

Jc1-Fluc HCVcc particles were produced as previously described [45]. Briefly, the pFKi389LucEiJFH1/J6/C-846 plasmid DNA was MluI (NEB, Ipswich, MA, USA) linearized and in vitro transcribed (IVT). 5 µg IVT RNA were electroporated in Huh-7.5.1 cells and incubated at 37 °C and 5% CO_2_. Virus supernatant was harvested 72 h after electroporation, filtered through a 0.45 µm pore filter (Carl Roth, Karlsruhe, Germany) and stored at −80 °C until further use. HCV P100pop was a kind gift from Professor Esteban Domingo (CBMSO, Madrid, Spain) [47].

To understand the impact of LLDs during HCV infection, 5 × 10^3^ Huh-7.5 or lipoprotein receptor-deficient cells were seeded in a 96-well plate and incubated for 24 h. The next day, cells were preincubated with selected LLDs 24 h before infection. Then, 50 µL virus and LLD were mixed and added to cells. 4 h post-infection (hpi), virus was removed and fresh DMEM with LLD was added. 72 hpi cells were lysed using an in-house lysis buffer (0.5 M Gly-Gly, 1 M MgSO_4_, 0.2 M EGTA, 1% Triton-X100) supplemented with DTT and frozen at −80 °C prior luciferase measurement. Luciferase assay was performed as described before [48] and measured on a Centro LB 960 microplate luminometer (Berthold technologies, Bad Wildbad, Germany).

### 2.5. HCV Kinetics in Primary Human Hepatocytes

Primary human hepatocytes (PHHs) from three separate donors were isolated from explanted livers and plated on collagen-coated 24-well plates with an approximate density of 3.75 × 10^5^ cells/well as previously described [49]. Informed consent was approved by the ethics commission of Hannover Medical School. One day after plating, culture media was changed to HCM media (Lonza, Basel, Switzerland) and treated with 1 µM Simvastatin, 5 µM Fenofibrate µM, 1 µM Alirocumab or DMSO (at the highest concentration used to dissolve the treatments). To control innate immune signaling, 10 µM ruxolitinib was also added to the cells (Adipogen Life Sciences, Liestal, Switzerland). Approximately 16 h after the pretreatment of the LLDs and ruxolitinib, the cells were infected with 3.75 × 10^5^ (measured on Huh-7.5 cells) of an HCVcc population with a high replication capacity (P100pop) [50,51]. 4 h later, the cells were washed with 5 × PBS and fresh media with drugs added. Supernatant and cells lysates were collected 4, 24, 72, and 96 h post-infection for quantification of intracellular HCV RNA and infectious particle release.

### 2.6. RNA Extraction and RT-qPCR Quantification of Viral RNA

To quantify intracellular HCV RNA in PHHs, total RNA from primary cells were isolated using a NucleoSpin RNA Kit (Macherey-Nagel, Düren, Germany) according to the manufacturer’s instructions. For each sample HCV RNA was quantified in technical replicates utilizing a LightCycler^®^ 480 RNA Master Hydrolysis Probes Kit (Roche Diagnostics, Rotkreuz, Switzerland) following the manufacturer’s instructions and the following primers and probe, 5′-UTR Forward primer, TCTGCGGAACCGGTGAGTA; 5′-UTR reverse primer, GGGCATAGAGTGGGTTTATCCA; and 5′-UTR probe, [6FAM]-AAAggACCCAgTCTTCCCggCAA-[TMR]. 10-fold serial dilutions of an in vitro-transcribed (IVT) HCV Jc1 RNA were used in each run for generation of a standard curve.

Viral RNA extraction and quantification of infected lipoprotein receptor knock-outs was based on previous work [52]. Briefly, HCVcc Jc1-WT or mock infected cells were lysed and RNA extracted according to Qiagen RNAeasy (Qiagen, Venlo, Netherlands) manufacturer’s protocol. Extracted RNA was measured using a NanodropTM 2000 (Thermo Fischer Scientific, Waltham, MA, USA). To detect viral RNA via RT-qPCR a probe against the HCV X-tail genomic region was used [53]. Each reaction comprised 3 µL of RNA and 17 µL of a mixture of Master Hydrolysis Probes (Roche, Basel, Switzerland), 300 nM HCMgR2 and 200 nM XFTF5 primers, and 100 nM HCVMGB2 custom probe. PCR was run for 45 cycles on a Light Cycler 480 thermocycler (Roche, Basel, Switzerland). 10-fold serial dilutions of IVT HCV Con1 RNA (pFKi 389 Neo EI Core 3’JFH1wt) were added in each run for generation of a standard curve. Absolute viral copy numbers were extrapolated from Ct values of samples versus the standard. Baseline and cut-off cycles were 3 and 40, respectively. Final copy numbers were normalized to the extracted total RNA concentration. Each biological sample was measured in duplicate.

### 2.7. TCID50 Assay

To quantify infectious particle release, 1 × 10^4^ cells/well of Huh-7.5 cells were seeded onto 96-well plates. One day after plating, 6 replicate wells were infected with HCV infected supernatant, serially diluted and incubated for 72 h. The cells were then fixed with 100% methanol for 10 min, followed by 2 washes with PBS plus 0.1% Tween-20 (Sigma–Aldrich, St. Luis, MO, USA) (PBS-T). Endogenous peroxidases were blocked with 3% H_2_O_2_ in PBS-T for 5 min. The cells were subsequently incubated with 440 pg/mL of anti-NS5A 9E10 antibody (Cell Essentials, Boston, MA, USA) at room temperature for 1 h. Unbound antibodies were washed twice with PBS-T and the cells were incubated 1 h with anti-mouse IgG-HRP (A4416, Sigma Aldrich, St. Louis, MO, USA) at a 1:300 dilution. Peroxidase activity was detected after 2 X PBS-T washing and the addition of a carbazole substrate (0.32% *w/v* 3-amino-9-ethylcarbazole in N,N-dimethylformamide diluted in 5 mM acetic acid, 10 mM sodium acetate, pH 8.0 and 0.4% H_2_O_2_) for 10 min. Unbound substrate was washed twice with H_2_O and stained cells were visualized under a microscope. Positive wells were counted and the tissue infective dose 50% (TCID50) was calculated using the Spearman and Kärber calculation [54].

### 2.8. Pseudoparticle Production and Transduction

Pseudoparticles for LLD treatments were generated as previously described [41]. Briefly, HEK293T producer cells were co-transfected with 1 µg DNA plasmids containing HIV gag-pol, envelope viral glycoproteins, and a NLuc or FLuc luciferase reporter gene in a 1:1:7 ratio using polyethylenimine (PEI) (Sigma–Aldrich, San Luis, MO, USA). 48 h and 72 h post-transfection, the pseudoparticles (pps) supernatant was collected, filtrated through a 0.45 µm pore filter (Carl Roth, Karlsruhe, Germany) and pooled together. Virus pp supernatant was stored at 4 °C for up to 7 days and at −80 °C for long term storage. For HCV GP-driven entry studies in lipoprotein receptor knock-out cell lines, HEK293T producer cells were co-transfected with 1.3 µg HIV gag-pol, 1.3 µg Fluc luciferase reporter gene, and 1 µg envelope GP with FUGENE^®^ HD reagent (Promega, Madison, WI, USA) (FUGENE 3:1 DNA) according to the manufacturer’s instructions.

To test the effect of LLDs on HCV GP-mediated entry, 5 × 10^3^ cells were seeded one day before in a 96-well plate and pre-incubated with different concentrations of LLDs an extra day before transduction. Polybrene (H9268-5G, Sigma–Aldrich, San Luis, MS, USA) was added to a final concentration of 4 µg/mL to 50 µL virus pps and applied together with LLDs or solvent controls. 6 h post-transduction (hpt) pps were removed and cells incubated for 72 hpt. Then, cells were lysed with 35 µL/well of our in-house lysis buffer (see previous) supplemented with DTT and frozen at −80 °C until measurement. For NLuc measurement, 20 µL lysate was mixed with 80 µL coelenterazine substrate (Carl Roth, Karlsruhe, Germany), incubated for 5 min at room temperature (rt) and measured as previously described [42] using a Centro LB 960 microplate luminometer (Berthold technologies, Bad Wildbad, Germany).

For assessment of HCV GP-driven entry in lipoprotein-deficient cell lines, 8 × 10^4^ cells/well were seeded using a 12 well format and incubated overnight at 37 °C and 5% CO_2_. 500 µL of pps encoding for Fluc were mixed with 4 µg/mL polybrene (see previous) and added to cells for 6 h. Viral supernatants were subsequently removed, and cells lysed 72 hpt. Fluc of 20 µL lysate was measured as previously described [55]. Specific GP-driven entry was determined as the differential luciferase signal from GP-bearing particles and no GP (NoEnv) negative control.

### 2.9. HCV Replication Assay

Subgenomic HCV (pFK-Luc-JFH1/ΔE1-E2 and pFK-Luc-JFH1/ΔE1-E2 ΔGDD) RNA was transcribed in vitro and transfected into the Huh-7.5 or lipoprotein receptor knock-outs by electroporation as previously described [45]. 1 × 10^5^ electroporated cells were seeded in 24-well plates for luciferase measurement and 10^4^ in 96-well plates for cell proliferation assessment. At the indicated time points, cells were either lysed using 200 µL of Fluc lysis buffer and frozen at −20 °C before measurement or assessed for cell proliferation using a MTT assay (MTT cell proliferation kit, OzBioSciences, Marseille, France). Fluc was measured as previously described using a Centro XS3 LB 960 plate luminometer (Berthold technologies, Bad Wildbad, Germany) [56] and proliferation with a Multiskan Go Microplate Spectrophotometer (Thermo Scientific, Waltham, MA, USA). After data collection, a FLuc/cell proliferation ratio was calculated for each time point and normalized to the ratio values at 4 h post-electroporation.

### 2.10. Cell Viability Measurements

To examine the effect of LLDs on cell viability, an MTT Assay was performed as previously described [57]. 0.5 mg/mL MTT substrate was added to drug-treated cells and incubated for 1 h at 37 °C. MTT substrate was then removed and DMSO was added to the cells. Absorbance at 570 nm was measured using a Biotek Synergy 2 ELISA plate reader (Winooski, VT, USA). Reference absorbance at 630 nm was subtracted from specific absorbance at 570 nm. Cells treated with MTT solution and 10% SDS were used as a negative control.

NLuc Assay was alternatively performed to analyze cell viability of LLDs during gp-mediated HCV entry experiments. To establish NLuc expressing Huh-7.5 cells, 30–50% confluent Huh-7.5 cells were transduced with VSV-Gpps carrying a NLuc reporter gene. After 6 h fresh DMEM complete medium was added. NLuc expression was evaluated three passages after transduction and at the end of each individual GP-mediated cell entry assay, as previously described [42].

### 2.11. Flow Cytometry

For quantification of extracellular protein expression, immunostaining with fluorescently-conjugated abs followed by flow cytometry was performed. In brief, adherent cells were washed with phosphate buffer saline (PBS), dislodged with non-enzymatic versene reagent (15040066, Gibco, Gaithersburg, MD, USA) and centrifuged at 1250 rounds per minute (rpm) for 5 min. Cell pellets were resuspended in FACS buffer (1% FCS in PBS) containing 1 µL FcR blocking reagent (Miltenyi Biotec Gmbh, Bergisch Gladbach, Germany) and transferred to a V-bottom 96-well plate (Sigma Aldrich, St. Louis, MO, USA). Subsequently, cells were either stained with saturating concentrations of conjugated mouse abs α-human APC CD81 (561958, BD Biosciences, Franklin Lakes, NJ, USA), α-human APC SR-B1 (363207, Biolegend, San Diego, CA, USA), and α-human PE LDLr (FAB2148P, Minneapolis, MN, USA) or their respective isotype controls for 20 min in the dark. Stained cells were washed once and resuspended in FACS buffer before analysis. Protein expression was quantified by APC or PE channel fluorescent intensity measurement using an Accuri C6 Plus flow cytometer (BD Biosciences, Franklin Lakes, NJ, USA). For quantification of endogenous levels of HCV entry co-factors, resuspended cells were fixed (1% FCS, 0.5% PFA in PBS) for 10 min ar RT, permeabilized (1% FCS, 0.1% Saponin in PBS) for 20 min on ice, and immunostained with monoclonal α-human occludin (0.4 µg/mL, mouse) (33–1500, Invitrogen/Thermo Scientific, Carlsbad, CA, USA), and polyclonal α-human claudin-1 (2 µg/mL, rabbit) (51–900) (Invitrogen/Thermo Scientific, Carlsbad, CA, USA) or α-human NPC1L1 (2 µg/mL, rabbit) (#5058, Cell Signaling Technology, Danvers, MA, USA) for 60 min on ice. Subsequently, cells were washed in FACS buffer and bound abs detected with 10 µg/mL Alexa Fluor 488 (AF488)-conjugated abs (goat α-mouse A-11029, goat α-mouse A-11034) (Invitrogen/Thermo Scientific, Carlsbad, CA, USA). Unspecific staining was controlled by immunostaining with equimolar concentrations of monoclonal mouse (555746, BD, Franklin Lakes, NJ, USA) or polyclonal rabbit IgG (I5006, Sigma Aldrich, St. Louis, MO, USA). Specific protein expression (ΔMFI) was calculated by subtracting the geometric mean intensity (MFI) of the isotype control staining from the specific ab MFI.

### 2.12. Western Blot

Expression of HCV entry factors was analyzed by protein immunoblot as previously described [55]. For protein detection, α-human SR-B1 (0.4 µg/mL, rabbit) (NB400-104, Novus Biologicals, Littleton, USA), α-human LDLr (1.34 µg/mL, rabbit) (ab52818, Abcam, Cambridge, UK), α-human occludin (1.25 µg/mL, mouse) (33–1500, Invitrogen/Thermo Scientific, Carlsbad, CA, USA), α-human claudin-1 (0.5 µg/mL, rabbit) (51–900, Invitrogen/Thermo Scientific, Carlsbad, CA, USA) or α-human NPC1L1 (0.25 µg/mL, rabbit) (#5058, Cell Signaling Technology, Danvers, MA, USA) were used. HRP-linked α-mouse (1:20,000) (A4416, Sigma Aldrich, St. Louis, MO, USA) and α-rabbit (1:20,000) (Jackson ImmunoResearch, Ely, UK) were used to detect proteins. For developing, HRP-based Amersham ECL Prime Western Blotting Detection Reagent (Cytiva Life Sciences, Marlborough, MD, USA) was used. Blots were documented with ChemoStar Professional Imager System (Intas Science Imaging Instruments, Göttingen, Germany).

### 2.13. Cholesterol Quantification Assay

Total cholesterol content of lipoprotein receptor-deficient cell lines was quantified using the Cholesterol Quantification Kit (MAK043) by Sigma Aldrich (St. Louis, Missouri, USA) according to the manufacturer’s instructions. Briefly, 1 × 10^6^ cells were collected and frozen at −20 °C until cholesterol quantification. Lipids were extracted using chloroform:isopropanol:IGEPAL CA-630 (7:11:0.1) and the organic phase was dried under vacuum using a Savant DNA-SpeedVac concentrator (Thermo Fischer Scientifc, Waltham, MA, USA). Extracted lipids were dissolved in Cholesterol Assay buffer and stored at −20 °C. Reaction mix was set up according to the manufacturer’s instructions and absorbance was measured at 570 nm in a Biotek Synergy 2 ELISA plate reader (Winooski, VT, USA). Cholesterol amount was extrapolated from an in-run cholesterol standard curve. Blank was subtracted from all values for background correction. The final cholesterol concentration was calculated by equating the cholesterol amount to the sample volume.

### 2.14. Lipid-Lowering Drugs

LLDs simvastatin (S6196) and fenofibrate (F6020) were purchased from Sigma Aldrich (St. Louis, MO, USA). Both compounds were diluted with DMSO to a final stock concentration of 50 mM and stored at 4 °C. Alirocumab (Praluent) was ordered from Sanofi (Paris, Île-de-France, FR) and was stored at 4 °C.

### 2.15. Patients and Methods

For this analysis 773 HCV infected patients who received antiviral treatment with DAA regimens at the outpatient clinic of Hannover Medical School from 2014 (approval of sofosbuvir)—2019 were considered. HCV RNA values, at specific time points during DAA therapy, from two different cohort subgroups were compared. In total, 15 patients did not show any documented HCV RNA values at the time of analysis and therefore were excluded. Patients with documented statin use at therapy start of DAA treatment were compared with patients without documented statin use at therapy start as control group. To adjust for possible confounders two matchings were performed. In matching one presence of cirrhosis and viral genotype were used as criteria to compare baseline values. In matching two, in addition to cirrhosis and viral genotype, HCV antiviral regimen was included to compare treatment values. The matching ratio was 2:1 generating a control group twice as big as the statin group. Matching partners were included in the analysis in the order of appearance in the original data set. We excluded patients without an appropriate matching partner. HCV RNA values were obtained at week 0, week 2, week 4, week 8, and week 12 using either the Cobas Ampliprep Cobas Taqman v.2.0 (Roche, limit of quantification 15 IU/mL) or the Aptima HCV Quant assay (Hologic; limit of quantification 10 IU/mL). If quantification limit for HCV RNA was reached (<15 IU/mL, <10 IU/mL) this was put into analysis as 15 IU/mL or 10 IU/mL. Undetectable HCV RNA was put into analysis as 1 due to logarithmic presentation of the data. Furthermore, clinical parameters, at baseline and/or treatment weeks, e.g., age, sex, HCV genotype, presence of cirrhosis, DAA therapy regimen, and AST as well as ALT levels were obtained. Clinical data were generated using standard laboratory procedures at Hannover Medical School. Cirrhosis was defined as fibroscan over 14.5 kPa. If fibroscan data were not available other clinical parameters for definition of cirrhosis were used. If no data were available for a particular time point, this dataset was excluded from the final analysis. Similar methods have been previously used by Maasoumy et al. [58].

### 2.16. Statistics

Infection, replication, and entry assays were performed three times (*n* = 3) with 3 technical replicates. Immunoblots were performed twice (*n* = 2). Cell surface and total stainings were carried out as 3 biological replicates. Cholesterol quantification was done thrice with two technical replicates. For the retrospective study, data was collected with Microsoft^®^ Excel^®^ 2010 (Redmond, WA, USA) and stated as absolute numbers, median and quartiles, always clearly labeled. Statistical analysis and visualization were done using GraphPad^®^ Prism^®^ 8 or 9 (La Jolla, CA, USA). Parametric, one, or two-way ANOVA corrected for multiple comparisons with Sidak or Dunnet methods as well as non-parametric unpaired Mann-Whitney U test were used to calculate statistical significance. *p*-value significance was represented as: * *p* ≤ 0.05; ** *p* ≤ 0.01; *** *p* ≤ 0.001; **** *p* ≤ 0.0001. *p*-values above 0.05 were considered not significant and were not illustrated.

### 2.17. Ethics

This analysis was performed according to the principles of good clinical practice as well as the declaration of Helsinki and approved by the local ethics committee (No. 2148-2014). All patients gave written informed consent.

## 3. Results

### 3.1. Glycoprotein-Driven HCV Entry Is Not Affected by Lipid-Lowering Drugs

**Figure 1:**
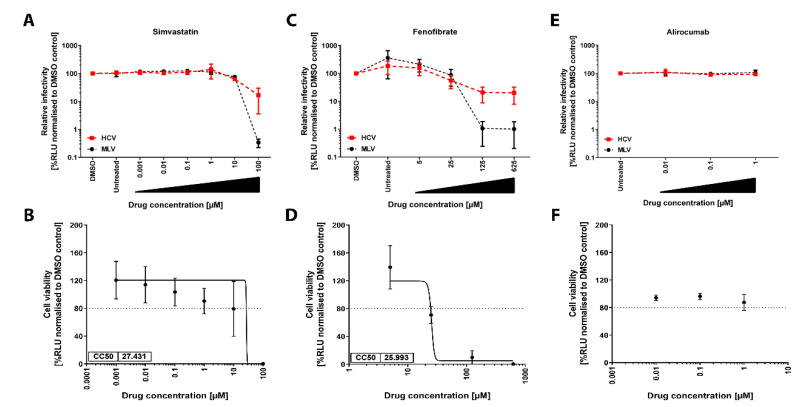
Lipid-lowering drugs (LLDs) do not affect Hepatitis C virus (HCV) genotype 1a entry into Huh-7.5 cells. Effect of LLD on HCV entry and cell viability. Dose-response of lentiviral pseudoparticle (pp) entry into Huh-7.5 (**A**,**C**,**E**). Cells were preincubated for 24 h with increasing concentrations of either simvastatin (**A**), fenofibrate (**C**), or alirocumab (**E**) before transduction with NLuc-encoding pps bearing E1-E2 GT1a glycoprotein (GP) (HCV), MLV env (MLV) for 6 h. NLuc activity was measured 72 h post-transduction (hpt) and luciferase activity was normalized to solvent control. MLVpp entry served as virus control. Drug-induced cytotoxicity (**B**,**D**,**F**). Huh-7.5 cells constitutively expressing NLuc (Huh-7.5/NLuc) were treated with either simvastatin (**B**), fenofibrate (**D**) or alirocumab (**F**) for 30 h as for (**B**,**D**,**F**) but were not transduced. Ninety-six h after treatment, NLuc activity was measured and luciferase activity values were normalized to DMSO control. When determined, CC50 values were indicated at the lower left side of the graph. Graphs represent the average of three independent experiments performed in triplicate with error bars representing the standard deviation (SD).

To examine the effect of lipid-lowering drugs (LLDs) on glycoprotein (GP) mediated HCV entry of hepatocytes, we pre-treated Huh-7.5 cells with increasing concentrations of simvastatin, fenofibrate and alirocumab for 24 h before transduction with pseudoparticles (pps) encoding for NLuc and pseudotyped with either HCV genotype 1a (H77) E1-E2 GPs (HCVpp), no GP (NoEnvpp) or Murine Leukemia Virus Env (MLVpp) in combination with drugs or solvent controls (Figure 1A,C,E). We used Huh-7.5/NLuc cells treated in parallel with the corresponding drugs to control for cell cytotoxicity (Figure 1B,D,F).

HCVpps and MLVpps transduced the cells about 10 to 400-fold above NoEnv levels, respectively (data not shown). The half-maximal inhibitory concentration (IC50) of simvastatin and fenofibrate in the HCVpp transduction assay was 10.2 µM and 25.3 µM, respectively (Figure 1A,C) and correlated with the inhibitory effects for MLV env-driven transduction (13.5 µM IC50 simvastatin, 25.9 µM fenofibrate), suggesting that the inhibitory effect is not HCV specific, but rather due to cell cytotoxicity. Indeed, the 50% cytotoxicity concentrations (CC50) determined in stably NLuc expressing cells of simvastatin and fenofibrate was 27.4 µM and 26.0 µM, respectively, strongly indicating that the antiviral effect of these compounds for HCV is associated to antiproliferative effects on Huh-7.5 cells (Figure 1B,D). The PCSK9 inhibitor alirocumab did not affect GP-driven entry or cell viability at the tested concentrations (Figure 1E,F). These results suggest that LLDs do not affect HCV GP-mediated cell entry at a non-toxic concentration.

### 3.2. Lipid-Lowering Drugs Do Not Impact HCV Infection of Human Immortalized or Primary Hepatocytes

**Figure 2:**
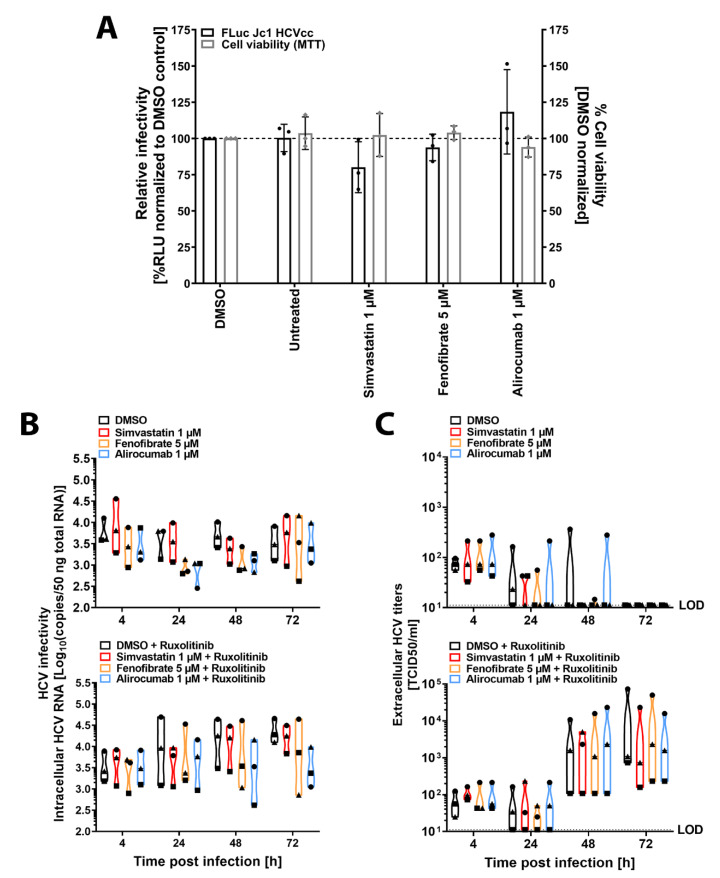
Lipid-lowering drugs do not affect HCV genotype 2a infection of Huh-7.5 or primary human hepatocytes. Cell culture-derived (HCVcc) infection (black bars) and viability (grey bars) in Huh-7.5 cells (**A**). Cells were pretreated for 24 h prior to infection with the indicated drug concentrations. Pretreated cells were infected with an FLuc reporter virus (Jc1-Fluc) in the presence of drug for 4 h. Virus inoculum was removed, and cells treated with LLDs for 68 h. FLuc activity was measured and normalized to solvent control (DMSO). Cell viability determined by MTT assay. Graph represents the average of 3 independent experiments with 3 technical replicates each. Dots show individual values of each experiment. Error bars represent the SD. For statistical analysis, a multiple comparison two-way ANOVA with a Dunnett correction was performed (α = 0.05, degrees of freedom (DF) = 20). HCVcc infection of primary human hepatocytes (PHH) (**B**,**C**). PHH from three donors were pretreated for 24 h with LLDs at the indicated concentrations in the absence (B-C, upper panels) or presence (**B**,**C**, lower panels) of the JAK/STAT inhibitor ruxolitinib (10 µM). Cells were then infected for 4 h with 3.75 × 10^5^ TCID50s of the GT2a cell-cultured adapted HCVcc p100pop in combination with the drugs. Virus inoculum was removed, and drugs added until the indicated times post-infection. Intracellular RNA copies (**B**) and extracellular infectious particles (**C**) were quantified by RT-qPCR and TCID50 assay, respectively. Graphs represent the distribution and median of each treatment of all donors. Each dot represents an individual donor (circle, triangle, square). Statistics were performed using a multiple comparison two-way ANOVA with a Dunnett correction (α = 0.05, degrees of freedom (DF) = 32).

Next, we set out to evaluate the impact of LLDs throughout the whole replication cycle. We selected the highest non-cytotoxic concentration of simvastatin (1 µM), fenofibrate (5 µM), and alirocumab (1 µM) (Figure 1B,D,F) and pre-treated Huh-7.5 cells before, during, and after HCVcc genotype 2a FLuc reporter virus (FLuc-Jc1) inoculation (Figure 2A). We run MTT assays in parallel to account for cell viability. All LLDs tested failed to significantly inhibit HCV infection. Merely, simvastatin showed numerical reduction in infectivity of 19.9%. Conversely, alirocumab mildly enhanced infectivity to 118.3% of control. LLDs did not affect cell viability as measured by the MTT assay. To validate these results in a more relevant model, we treated primary human hepatocytes (PHH) from three different donors with the selected LLDs alone or in combination with the JAK/STAT pathway inhibitor ruxolitinib and infected them with p100pop, a cell culture-adapted HCVcc virus with higher replication fitness (Figure 2B,C) [47,50,51]. As expected, immunocompetent cells partially (48 hpi) or completely (72 hpi) controlled HCV particle release and ruxolitinib treatment circumvented this restriction (Figure 2C). Importantly, LLDs did not significantly affect HCV replication (Figure 2B) or release (Figure 2C) at any of the time points tested or when the innate immune response was active or blunted. Altogether, our results suggest that these drugs do not significantly affect HCV infection in human hepatoma cells.

### 3.3. Genetic Ablation of Lipoprotein Receptors SR-B1 and LDLr Does Not Affect HCV Entry Factor Expression or Cholesterol Content

**Figure 3:**
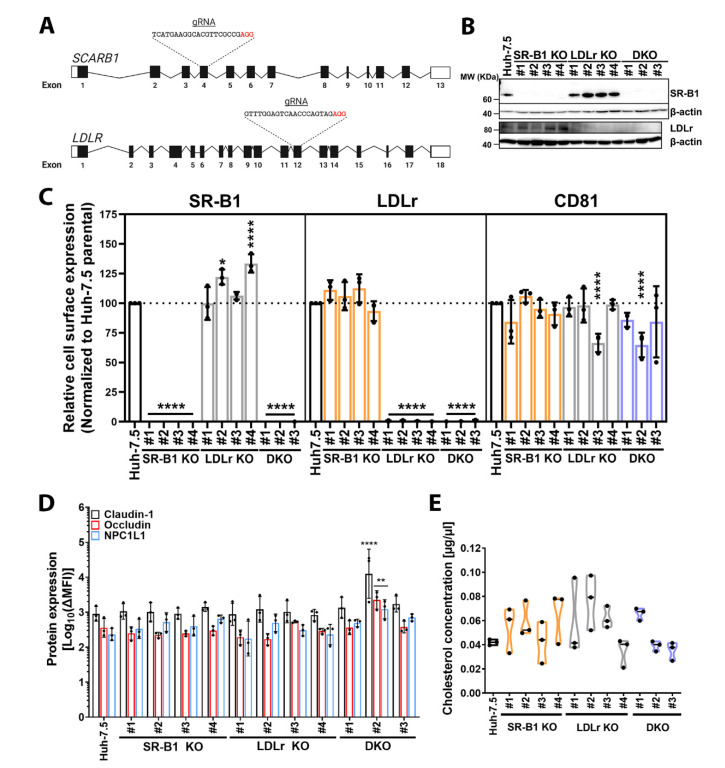
Characterization of lipoprotein receptor-deficient cell lines. Lipoprotein receptor knock-out (KO) strategy and characterization. Lipoprotein receptor *SCARB1* (SR-B1) and *LDLR* (LDLr) gene scheme (**A**). Graphic representation of genes for SR-B1 and LDLr. Exons are shown as boxes with black areas representing open reading frames (ORFs). Introns are shown as lines. CRISPR-Cas9 guide RNA (gRNA) nucleotide sequence targeting *SCARB1* and *LDLR* are depicted. Protospacer adjacent motif (PAM) is highlighted in red. Image created with BioRender.com (accessed 09/04/2020). Endogenous expression of lipoprotein receptors SR-B1 and LDLr (**B**). Cell lysates were evaluated for expression of indicated proteins by immunoblot using abs against the specific targets. β-actin was used as an internal control (42 kDa). Cell surface expression of SR-B1, LDLr, and CD81 in SR-B1 KO clones (light orange bars), LDLr KO clones (grey bars), and DKO clones (light blue bars) (**C**). Saturating concentrations of fluorophore conjugates abs against cellular targets and their isotype controls were used to stain cells for 20 min at 4 °C. Stained cells were analyzed by flow cytometry and ΔMFI was calculated as stated in the methods. ΔMFI values were normalized to parental Huh-7.5 cells. Endogenous expression of HCV entry factors claudin-1, occludin, and NPC1L1 (**D**). Protein expression was determined by immunostaining of permeabilized cells with equal amounts of specific abs or isotype controls for 60 min at 4 °C, followed by detection of bound abs with an Alexa Fluor 488 (AF488)-conjugated secondary ab. Specific staining was determined as ΔMFI. Total cholesterol in lipoprotein receptor KOs (**E**). Lipids were extracted from cells as described in the methods. The cholesterol fraction was measured using a colorimetric assay and cholesterol amount extrapolated from an in-run standard curve and transformed to cholesterol concentration (µg/µL). KO cells are shown in the same color patterns as for C. Graphs C, D, and E display the average of three experiments performed in triplicate and represent individual values as black dots. SD is shown as error bars. Statistical significance of the total (**D**) or cell surface staining (**C**) was assessed by a two-way ANOVA with a Dunnett post hoc (α = 0.05, DF = 72) and with a one-way ANOVA corrected with Dunnett post hoc for the cholesterol content (α = 0.05, DF = 24) (**E**). Statistical significance is shown as *p*-value: * *p* ≤ 0.05; ** *p* ≤ 0.01; **** *p* ≤ 0.0001.

To understand the role of lipids and cholesterol homeostasis in HCV infection in more depth we first decided to block Huh-7.5 cell lipid, and specifically cholesterol, uptake by genetic ablation of the two main receptors for LDL (LDLr) and HDL (SR-B1) separately or in combination by CRISPR-Cas9 technology. We targeted exon 4 of the *SCARB1* SR-B1 gene and exon 12 of the LDLr gene (*LDLR*) since these exons are essential for transcription and translation of all known isoforms of these two proteins (Figure 3A). We generated and characterized 4 individual clones for SR-B1 (#1, #2, #3, #4) and LDLr (#1, #2, #3, #4) KOs as well as three clones (#1, #2, #3) for SR-B1/LDLr DKOs.

We confirmed that the corresponding clones did not express SR-B1 and/or LDLr by immunoblot (Figure 3B). These results were also confirmed for cell surface expression of the respective proteins by flow cytometry (Figure 3C). Interestingly, SR-B1 cell surface levels in LDLr KO clones #2 and #4 significantly increased in comparison to Huh-7.5 parental cells but LDLr cell surface expression did not increase in any of the SR-B1-deficient clones.

To rule out compensatory effects, we next assessed the surface expression of HCV entry factor CD81 [59] (Figure 3C, right panel) as well as the total expression of entry co-factors claudin-1, occludin, and Niemann-Pick C1-Like 1 (NPC1L1) [41,60,61] in lipoprotein receptor-deficient cells (Figure 3D and Figure S1B). Notably, we observed a significant decrease of CD81 surface expression levels in LDLr KO clones #3 and DKO clone #2. Claudin-1, occludin and NPC1L1 were detected by immunoblot at the expected sizes of 22, 65 and 140 kDa, respectively, in all clones [Figure S1]. In contrast to CD81, we did not observe any drastic change in protein expression levels of claudin-1, occludin, and NPC1L1 in all KO clones but DKO #2, which significantly increased expression of all three proteins (Figure 3D). Finally, we quantified cholesterol content to rule out any possible secondary effect on HCV infection due to alteration of cellular cholesterol content. We did not find any statistically significant differences in cholesterol content among the KO clones regardless of their genetic background (Figure 3E). The median cholesterol concentrations in lipoprotein receptor KO clones were slightly higher or similar to parental Huh-7.5 cells (Figure 3E).

In summary, these results suggest, that genetic ablation of SR-B1 and/or LDLr does not drastically impact HCV entry factor expression or cellular cholesterol content.

### 3.4. LDLr and SR-B1 Play a Redundant Role in HCV Infection but Not in Entry

**Figure 4:**
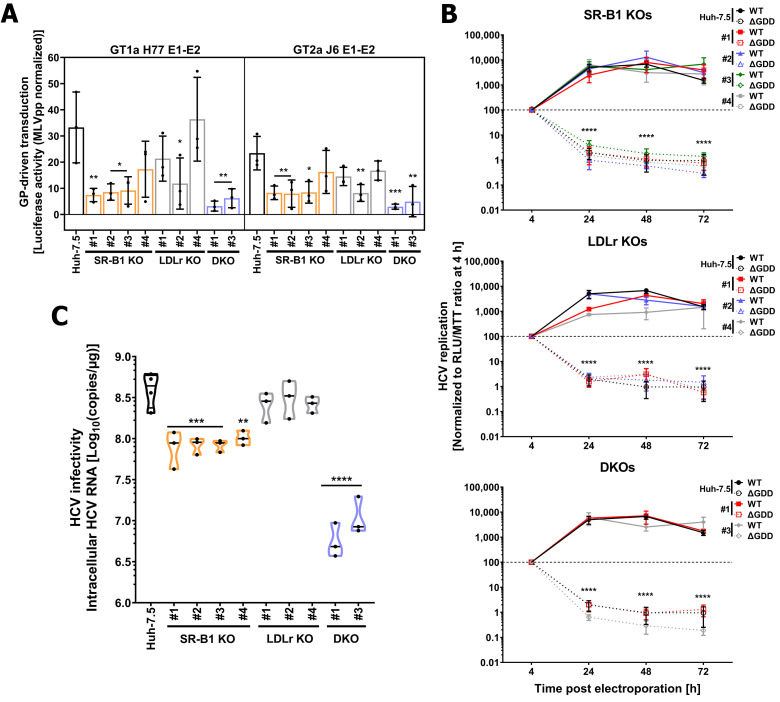
Lipoprotein receptor SR-B1 and LDLr do not affect HCV replication, SR-B1 facilitates GP-driven cell entry, and both redundantly participate in HCV infection. HCV GP-mediated entry in lipoprotein-deficient cells (**A**). Huh-7.5 parental (black bars), SR-B1 (light orange bars), LDLr (grey bars), and DKOs (light blue bars) were transduced with NLuc-encoding GT1a H77 or GT2a J6 HCVpp, negative (NoEnvpp), and positive (MLVpp) controls for 6 h. Six hpt, lentiviral particles were removed and reporter expression measured 72 h after. Raw relative light units (RLU) values were normalized to MLVpp values of the same cell line to account for intercellular variation. NoEnvpp normalized values were subtracted to represent HCV GP-specific entry. HCV subgenomic replication in Huh-7.5 and lipoprotein receptor-deficient cells (**B**). Cells were transfected with a HCV GT2a replication-competent (WT) or deficient (ΔGDD) subgenomic genomes tagged with FLuc. RLU values were normalized to 4 h post-electroporation and to MTT values to account for initial RNA delivery and cell growth variabilities between cell lines. Graph represents the average of three independent experiments performed in triplicate +/− sem. HCVcc infection of lipoprotein-deficient and parental cells (**C**). Indicated cell lines were infected untagged GT2a Jc1 HCVcc. Virus inoculum was removed hpi. Twenty-four hpi cells were lysed and Intracellular HCV RNA copies determined with an X-tail based absolute RT-qPCR. Data are shown as violin plots with median (black line) and individual values (black dots). Data distribution is symbolized by the plots’ shape. A two-way ANOVA with Dunnett post hoc (α = 0.05, DF = 10) was used for statistical analysis of HCV entry and replication assays and a one-way ANOVA with Sidak’s post hoc for HCVcc infection assay (α = 0.05, DF = 25). *p*-value was calculated and shown as: * *p* ≤ 0.05; ** *p* ≤ 0.01; *** *p* ≤ 0.001; **** *p* ≤ 0.0001.

To delineate the role of each lipoprotein receptor in HCV infection, we next evaluated the susceptibility and permissiveness of lipoprotein receptor KOs to HCV. We excluded LDLr KO #3 and DKO #2 from our main analysis due to their decreased expression of surface CD81 (Figure 3C). We transduced KO and DKO cells with FLuc-encoding NoEnvpp, HCVpp from GT1a (H77) and GT2a (J6), or MLVpps for 6 h and measured FLuc activity 72 h later. We set MLVpp luciferase activity of each experiment to 100 percent (data not shown), normalized each virus to this value, and subtracted the unspecific entry of NoEnvpps (Figure 4A). Three out of four SR-B1 KO clones were significantly less susceptible to HCV GP-driven entry regardless of their genotype. Conversely, only LDLr KO #2 had lower susceptibility to HCV GP-mediated entry. Importantly, both DKO clones showed significantly lower HCVpp entry. The average fold-reduction for GT1a H77 HCVpp among lipoprotein receptor KO cells was 3.5 for SR-B1 and 1.8 for LDLr clones, and 2.5 and 2 for GT2a J6 in SR-B1 and LDLr clones, respectively. Finally, the average HCVpp entry was 7.9-fold lower for GT1a H77 pps and 6.3 for GT2a J6. Notably, statistical analysis did not show any significant differences in HCVpp susceptibility between SR-B1 and DKOs. LDLr KO #3 and DKO clone #2 were highly refractory to HCVpps, with LDLr KO #3 being virtually resistant to HCV GT2a J6 E1-E2 transduction [Supplemental 2A]. Our results confirm the previously observed role of SR-B1 cell surface expression for HCV GP-driven cell entry and suggest a minor synergistic effect of SR-B1 and LDLr. 

LDLr has previously been implicated in HCV replication [15]. To determine the role of lipoprotein receptors in this step of the virus life cylce, we electroporated Huh-7.5 or KO cells with a replicative GT2a HCV subgenomic RNA (WT) or a replication-deficient subgenomic RNA (ΔGDD) that encodes for Fluc (Figure 4B and Figure S2B). As expected, all SR-B1 KOs replicated at similar rates as Huh-7.5 (Figure 4B). Interestingly, while LDLr KOs #1 and #4 had lower replication rates at 24 and #4 at 48 h post-electroporation, all DKOs tested had similar replication rates (Figure 4B and Figure S2B). These results suggest a clone rather than protein-specific effect. As expected, replication-deficient ΔGDD failed to replicate in all cell lines tested (Figure 4B and Figure S2B). These results rule out a possible effect of lipoprotein receptors in HCV replication.

Lastly, we inoculated all KO and parental Huh-7.5 cells with untagged Jc1 GT2a HCVcc for 4 h and let the infection progress for 20 h (Figure 4C and Figure S2C). LDLr ablation did not affect HCV infectivity in all clones (Figure 4C) but #3 (Figure S2). In line with the HCVpp results (Figure 4A), infectivity was significantly reduced in all SR-B1 KO clones with an average 4.8-fold reduction of HCV copies in comparison with parental Huh-7.5 cells. Strikingly, cell permissiveness was greatly reduced in all DKOs (approx. 52-fold) compared to parental cells (Figure 4C and Figure S2C). These data suggest that lipoprotein receptors redundantly promote HCVcc infection.

### 3.5. Lipid-Lowering Drugs Affect HCV Infection in Lipoprotein Receptor-Deficient Cell Lines

To investigate how cellular lipid homeostasis and its modulation by LLDs impacts HCV infection in a genetically altered background, we selected SR-B1 KO #3, LDLr KO #1, and DKO #1 for treatment with selected LLDs.

**Figure 5:**
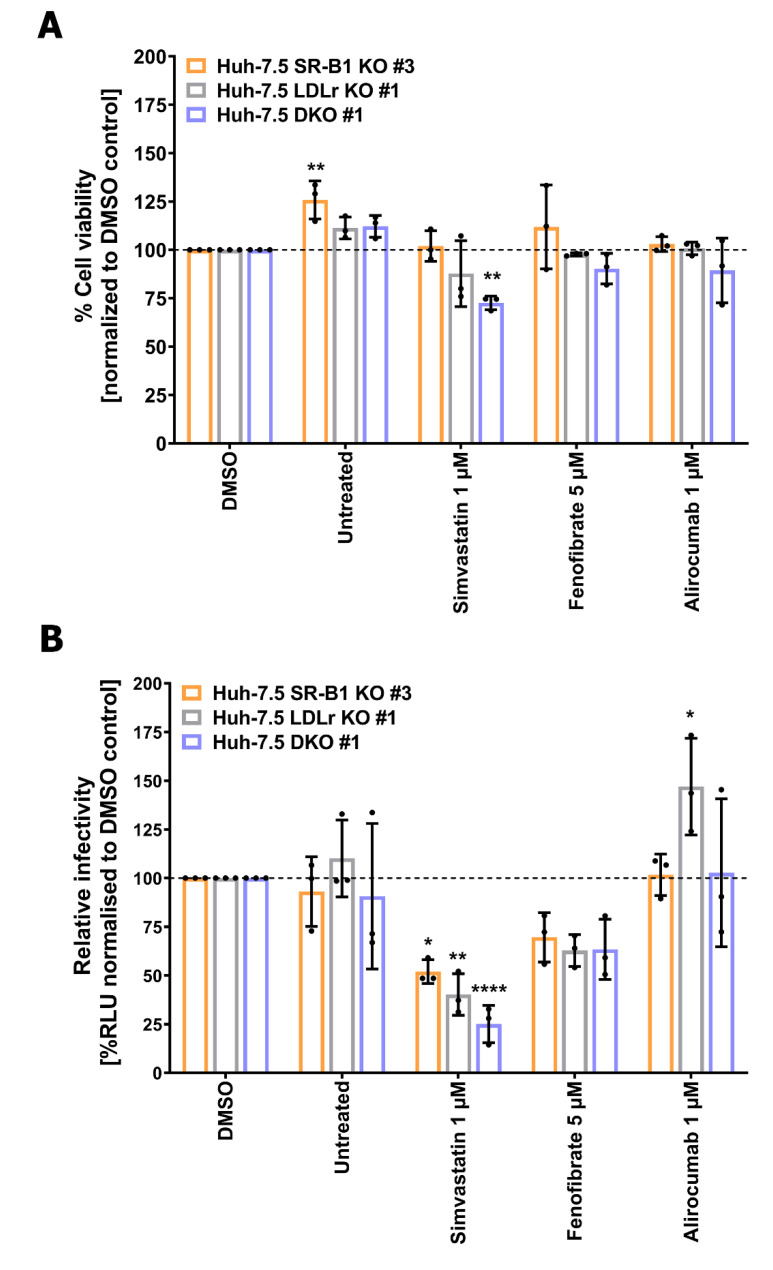
Lipid-lowering drugs affect HCV infection of lipoprotein receptor-deficient cell lines. Drug-induced cell cytotoxicity of lipoprotein receptor-deficient cell lines (**A**). Huh-7.5 derived SR-B1 KO #3 (light orange bars). LDLr KO #1 (grey bars) and DKO #1 (light blue bars) were treated with 1 µM simvastatin, 5 µM fenofibrate or 1 µM alirocumab for 96 h. Cell viability was determined by MTT Assay. (**B**) HCVcc infection of KO cells (**B**). SR-B1 KO #3, LDLr KO #1, and DKO #1 were treated with indicated drugs 24 h before infection with an FLuc reporter GT2a HCV virus (Fluc-Jc1) in the presence of the compounds or solvent control. 4 h later, virus was removed and drugs applied for extra 72 h before cell lysis and FLuc measurement. FLuc activities were normalized to DMSO control. Graphs represent 3 independent experiments performed in triplicates. Bars show the average and error bars in SD. Black dots plot individual values. Multiple comparison two-way ANOVA with a post hoc Dunnett correction was performed for statistical analysis (α = 0.05, DF = 40). * *p* ≤ 0.05; ** *p* ≤ 0.01; **** *p* ≤ 0.0001.

First, we tested whether LLDs could have a cytotoxic effect on cells devoid of lipoprotein receptors. Therefore, we treated each lipoprotein-deficient cell line with the concentrations previously tested on Huh-7.5 cells (1 µM simvastatin, 5 µM fenofibrate, 1 µM alirocumab) for 96 h before performing an MTT-based cell viability assay (Figure 5A). Whereas alirocumab and fenofibrate did not induce cytotoxicity in any of the clones, simvastatin moderately but significantly reduced cell viability by 27.39% when both proteins were lacking.

We next tested the effect of LLDs on HCV infectivity in the lipoprotein-deficient cells in a similar fashion as for Huh-7.5 parental cells (Figure 2). FLuc activity values were measured 72 hpi and normalized to solvent control (Figure 5B). Strikingly, simvastatin and fenofibrate reduced HCV infection in all KO cell lines. Although fenofibrate diminished HCV in all KO cell lines by approx. 30-40%, this reduction was not statistically significant. Strikingly, simvastatin significantly inhibited HCV infection differentially in the three KOs: single SR-B1 and LDLr KOs had a residual infectivity of 52% and 40%, respectively, but the effect was additive when both lipoproteins were lacking (25% residual HCV infection), suggesting that de novo cholesterol synthesis is important for HCV infection. Finally, alirocumab showed a slightly increased infectivity in LDLr KO #1 cells that was not seen in the other clones tested. Thus, it appears that among the tested drugs, statins can have a modest inhibitory effect on HCV infectivity in vitro when SR-B1 and/or LDLr are ablated.

### 3.6. Retrospective In-Vivo Analysis of a HCV Patient Cohort Suggests a Slight Beneficial Effect of Statin Usage on Liver Health

**Figure 6:**
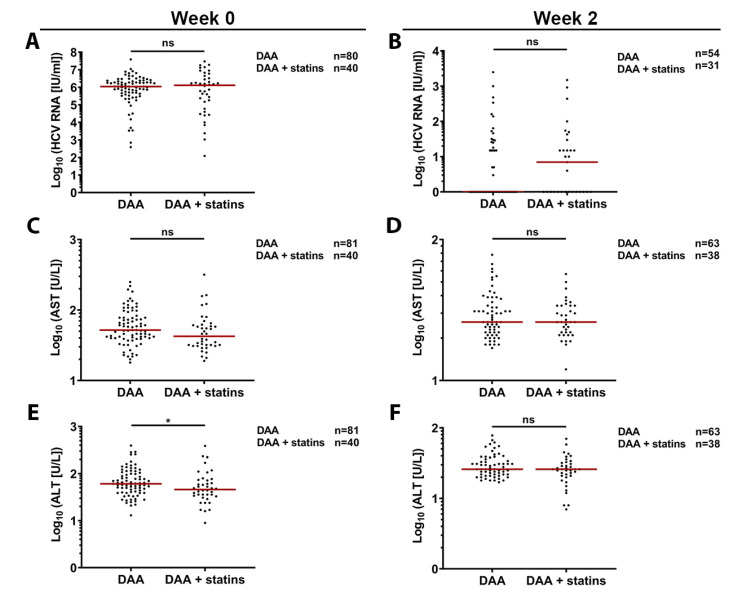
Mildly decreased aspartate aminotransferase (AST) and alanine aminotransferase (ALT) levels before HCV therapy in chronically infected patients taking statins. HCV RNA blood levels in patients following HCV direct-acting antiviral (DAA) therapy regimens in the presence or absence of statins (**A**,**B**). AST (**C**,**D**) and ALT (**E**,**F**) blood levels during HCV DAA treatment. Number of patients included in each analysis is shown in the legend and data points for each patient are represented as individual dots. For statistical analysis unpaired, two-tailed, no parametric Mann-Whitney test was performed. No significant (ns) *p* > 0.05; * *p* ≤ 0.05.

Finally, we retrospectively analyzed a cohort of 717 chronically HCV infected patients and compared statin users and non-statin users in terms of liver damage and HCV RNA value decline throughout a certain part of the treatment period (week 0 and 2). Table S1 shows baseline characteristics of the analyzed cohort. In line with previous surveys in Germany [60], approx. 5.72% of chronically infected patients were receiving a statin at the start of antiviral treatment. Importantly, we could see differences between the two study groups such as different frequencies of cirrhosis and genotype 3 infection or inconstant distribution of HCV regimens. To adjust for confounders like presence of cirrhosis, viral genotypes, and used HCV regimens, we performed two different matchings. Matching one includes cirrhosis and genotype while matching two additionally covers HCV regimen. Baseline characteristics of the compared groups are shown in Tables S2 and S3. We used matching one (GT, cirrhosis) to evaluate viral RNA (Figure 6A), aspartate aminotransferase (AST) (Figure 6C), and alanine aminotransferase (ALT) (Figure 6E) levels prior to start of the HCV therapy. Although we found no differences in RNA levels (Figure 6A) between patients, we observed a lower trend for AST levels (52 U/L for DAA vs. 42.5 for DAA + statins) (Figure 6C) and a statistically significant reduction for ALTs values (61 U/L vs. 46) in patients under statin treatment (Figure 6D). Two weeks after beginning HCV treatment, we accounted for cirrhosis, GT, and HCV regimen (matching two) and observed no significant differences in viral RNA, AST, or ALT levels (Figure 6B,E,D). Taken together, our results indicate that statins do not likely affect HCV infection in vivo but may partially protect chronically infected patients before therapy from virus-associated liver damage.

## 4. Discussion

The well-established link between lipid metabolism and HCV replication cycle prompted us to study the interplay between lipoprotein receptors and lipid-lowering drugs (LLDs) during HCV infection. While some aspects of the interaction between the HCV replication cycle and LLDs—most notably statins—have previously been studied by others, it remained elusive if combined genetic and pharmacological perturbations targeting host lipid metabolism affect HCV replication. We could confirm that lipoprotein receptors SR-B1 and LDLr play a redundant role in HCV infection that is not dependent on cellular cholesterol content or expression of HCV entry co-factors. Furthermore, we could demonstrate that HCV is more susceptible to LLDs simvastatin and fenofibrate when these receptors are missing. Lastly, statins do not seem to influence HCV viral loads but may improve liver health in vivo in our retrospectively analyzed cohort of HCV infected patients under antiviral therapy.

It is widely accepted that the lipoprotein receptor SR-B1 is critical for HCV entry, while the role of LDLr and whether it is truly required for HCV entry has long been debated. SR-B1 is known to interact with both LVP-embedded ApoE and the viral glycoprotein E2, which ultimately facilitate binding of E2 to the CD81 receptor [11,14]. Moreover, naturally occurring single-nucleotide polymorphisms (SNPs) S112F and T175A are both associated to high HDL-cholesterol serum levels and decrease HCV uptake [13,61]. Here we showed that ablation of SR-B1 affects GP-driven entry, confirming these observations. Likewise, LDLr promotes viral entry by a process dependent on its lipid-binding region [12,13] and an association between SNP rs688 and HCV resistance among HCV multiple exposed individuals has been suggested [62]. In 2016, Yamamoto and colleagues showed that SR-B1 and LDLr may redundantly participate in HCV entry and this effect was dependent on the HDL and LDL binding regions of SR-B1 and LDLr, respectively [13]. However, expression of HCV entry factors such as occludin, claudin-1, or NPC1L as well as differences in cellular cholesterol content were not assessed in the lipoprotein receptor-deficient cell lines in this previous study. Our study confirms and builds on their results by showing that HCV co-factors and cholesterol content are not dramatically affected by lipoprotein receptor ablation (Figure 3D,E). Notably, we observed interclonal variation of SR-B1 and CD81 cell surface levels (Figure 3C). Alterations in CD81 surface expression levels has previously been reported in clonal populations of hepatoma cells [63,64]. Whereas SR-B1 upregulation in LDLr knock-out (KOs) was mild, decreased CD81 cell surface expression was substantial in some clones and this may disturb HCV uptake. In keeping with this, HCV entry and infection in the clones LDLr KO #3 and DKO #2 was greatly reduced (Figure S2A,C). These results bring to light the drawbacks of the genetic variability among KO cell clones sourcing from a common parental cell line and underscore the importance of analyzing multiple KO clones for conclusive findings.

LLDs failed to decrease HCV infection in Huh-7.5 or PHH cells in our study (Figure 2). Although this is in agreement with previous research regarding the PCSK-9 inhibitor alirocumab (31), our results differ from previous findings for the statin simvastatin [27,28]. This disparity might be related to drug-induced cytotoxicity since drug concentrations used in previous studies were close to our calculated half-maximal cytotoxic concentration (CC50, 27.4 µM, Figure 1). Alternatively, known pleiotropic effects of statins [reviewed in [65,66]] might differ depending on the cell type. Finally, to our knowledge, this is the first study investigating the role of fenofibrate on the whole HCV replication cycle. In summary, we suggest that clinically-relevant drug concentrations [67] of the selected LLDs do not affect HCV infection.

Statins target 3-hydroxy-3-methyl-glutaryl-coenzyme A (HMG-CoA) reductase, a rate-limiting, pivotal enzyme of the mevalonate pathway [19,20]. This pathway controls both de novo cholesterol synthesis and protein prenylation (geranylgeranylation and farnesylation) [20]. For HCV it was previously suggested that replication is dependent on geranylgeranylation of an unknown host protein [26]. Here we showed in lipoprotein receptor-deficient cell lines (Figure 3A,B) that statins significantly decrease HCV infection (Figure 5B) without a major impact on cell viability (Figure 5A). This suggests de novo cholesterol synthesis and not protein prenylation as the main determinant of HCV susceptibility (Figure 5B). Nevertheless, additional effects of statins on HCV replication via protein prenylation might explain the slight reduction we observed in parental Huh-7.5 cells after treatment with simvastatin (Figure 3A). Future studies should address which steps of the HCV replication cycle are modulated in lipoprotein-deficient cells after statin treatment as well as which metabolites (mevalonate, geranylgeranyl pyrophosphate, and squalene) could rescue HCV infectivity.

Alirocumab targets the secreted proprotein convertase subtilisin/kexin type 9 (PCSK-9), which binds to LDLr at the plasma membrane and promotes its lysosomal degradation [23]. We observed a slight enhancing effect of alirocumab on HCV infectivity in the LDL receptor-deficient cells (Figure 5B). Although we do not expect this effect to be biologically relevant, we hypothesize that in the absence of LDLr, PCSK-9 might bind surface SR-B1 and target it for degradation, as previously suggested [68]. Therefore, alirocumab may prevent the interaction of PCSK-9 and SR-B1 and leave SR-B1 cell surface levels unaltered. Following studies could address this theory by assessing the cell surface levels of SR-B1 in the presence or absence of alirocumab in LDLr-deficient cell lines. Lastly, fibrates stimulate peroxisome proliferator-activated receptor (PPAR) alpha, which in turn inhibit triglyceride synthesis and increase lipoprotein lipase activity and thereby leading to decreased VLDL assembly and secretion [22]. Fenofibrate mildly inhibited HCV infection of all tested lipoprotein-deficient cell (Figure 5B). Interestingly, reduction of HCV infection was comparable in all cell lines regardless of their genetic background, suggesting that the mode of action of fenofibrate is common in all lipoprotein receptor-deficient cells. Since fibrates induce fatty acid uptake [69] and reduce VLDL secretion [70], ablation of lipoprotein receptors preventing fatty acid uptake together with fibrate treatment decreasing VLDL synthesis results in decreased HCV assembly and release. On the other hand, lipoprotein lipase has been shown to block HCV entry [71]. An increase of this enzyme by fibrates may thus have an additional inhibitory effect. Taken together, we observe differential effects of different classes of LLDs on HCV infection.

The effect of statins on HCV infection in patients is controversial [29,35,36]. A study on recently diagnosed chronically HCV infected patients did not see differences in viral loads [35]. Conversely, combination of statins with PEGylated interferon or direct-acting antivirals (DAAs) significantly decreased HCV viral loads and improved sustained virological responses among patients as compared to PEGylated interferon or DAA treatment alone [29,36]. Our findings suggest that statins may not have a beneficial effect when in combination with specific HCV therapeutics (Figure 6 B,D,F). Besides their role as LLDs, statins are also known to be anti-inflammatory, antifibrotic, and antiangiogenic and thus are thought to protect against chronic inflammatory diseases such as progression of hepatic fibrosis [reviewed in [72]]. Our findings may partly support this model showing a lower trend of AST and significantly reduced ALT levels in patients under statin therapy before DAA treatment (Figure 6C–F). The most important limitation of the studied cohort is the heterogeneous nature of the baseline characteristics. Indeed, different frequencies of cirrhosis and genotype 3 infections, as well as the inconstant distribution of the HCV regimens between the groups can be seen as confounding factors. Hence, we adjusted for possible confounders by matching for different parameters. Matching two (cirrhosis, genotype and regimen) is in our opinion superior to matching one (cirrhosis, genotype) when it comes to interpreting the time points after therapy start. Matching one should only be used to especially focus on week 0 data due to the missing regimen matching. Therefore, significant differences in RNA levels and liver damage markers after therapy start should be neglected in this case. Additionally, the analysis has more limitations. No dosing data of the statins were included, and statin use was only evaluated at one time point during the analysis. Due to the fact that the matching was done in the order of appearance in the original data set, this type of matching could lead to different results when choosing different matching partners from the original cohort. Nevertheless, we think that these in vivo data may add some interesting hints to the mainly experimental dimensions of this work, but we suggest a careful interpretation of the data.

In summary, in this study, we comprehensively analyzed the combined effects of LLD treatment and genetic alterations of lipoprotein receptors on HCV infection. Our findings suggest that fibrates and PCSK-9 inhibitors do not considerably impact HCV infectivity, whereas statins can affect the HCV replication cycle by inhibition of the de novo cholesterol synthesis branch of the mevalonate pathway. Importantly, statins may partly protect against virus-induced liver damage. Future studies dissecting the HCV replication cycle steps affected by statins may shed light on the specific mechanism underlying statin-induced HCV inhibition.

## Data Availability

The data presented in this study are available on request from the corresponding author. The data are not publicly available due to patient confidentiality.

## Supplementary Materials

The following are available online at https://www.mdpi.com/article/10.3390/cells10071626/s1, Figure S1. HCV entry co-factor expression. Figure S2. HCV entry, replication, and infectivity of lipoprotein receptor knock-outs LDLr #3 and DKO #2. Table S1. Baseline characteristics of patient cohort. Table S2. Matching 1 characteristics of patients. Table S3. Matching 2 characteristicis of patients.
