# Supplementary material for: Interdependent Impact of Lipoprotein Receptors and Lipid-Lowering Drugs on HCV Infectivity
Source: Cells. 2021 Jun 29;10(7):1626. doi: 10.3390/cells10071626 (PMC8303410; doi:10.3390/cells10071626)
Supplement: Supplementary file 1 [file cells-10-01626-s001.zip › Table S1.pdf]

Table S1. Characteristics of patients.

	patients with statin use at TS	patients without statin use at TS
**number of patients [n]**	**41**	**717**
age [years; median, range]	63 (31-80)	55 (18-85)
sex [n, %]		
male	23 (56.1)	401 (55.9)
female	18 (43.9)	316 (44.1)
genotype [n, %]		
1	30 (73.2)	501 (69.9)
1/2	0 (0)	1 (0.1)
1/4	0 (0)	2 (0.3)
1/6	1 (2.4)	2 (0.3)
2	7 (17.1)	26 (3.6)
2/4	0 (0)	1 (0.3)
3	1 (2.4)	143 (19.9)
4	0 (0)	23 (3.2)
5	1 (2.4)	6 (0.8)
6	0 (0)	1 (0.3)
n/a	1 (2.4)	11 (1.5)
used HCV regimen [n, %]		
ELB/GRZ	15 (36.6)	90 (12.6)
SOF/LDV±RBV	10 (24.4)	189 (26.4)
SOF/RBV±PEG-IFN	4 (9.8)	104 (14.5)
GLE/PIB	3 (7.3)	67 (9.3)
VEL/SOF±RBV	3 (7.3)	85 (11.9)
OMB/PTV/r/DSV±RBV	3 (7.3)	82 (11.4)
OMB/PTV/r±RBV	0 (0)	7 (1.0)
SOF/DAC±RBV	1 (2.4)	43 (6.0)
SOF/SIM±RBV	1 (2.4)	37 (5.2)
VEL/SOF/VOX	1 (2.4)	12 (1.7)
SIM/RBV/PEG-IFN	0 (0)	1 (0.1)
cirrhosis [n, %]	9 (22.0)	281 (39.2)
n/a	0 (0)	8 (1.1)
statins [n, %]		
simvastatin	16 (39.0)	0 (0)
pravastatin	10 (24.4)	0 (0)
atorvastatin	9 (22.0)	0 (0)
fluvastatin	6 (14.6)	0 (0)

TS therapy start, n/a not applicable, HCV hepatitis C virus, OMB ombitasvir, PTV paritaprevir, r ritonavir, DSV dasabuvir, ELB elbasvir, GRZ grazoprevir, VEL velpatasvir, SOF sofosbuvir, GLE glecaprevir, PIB pibrentasvir, DAC daclatasvir, LDV ledipasvir, RBV ribavirin, SIM simeprevir, VOX voxilaprevir, PEG-IFN pegylated interferon.
