# Supplementary material for: Interdependent Impact of Lipoprotein Receptors and Lipid-Lowering Drugs on HCV Infectivity
Source: Cells. 2021 Jun 29;10(7):1626. doi: 10.3390/cells10071626 (PMC8303410; doi:10.3390/cells10071626)
Supplement: Supplementary file 1 [file cells-10-01626-s001.zip › Table S2.pdf]

Table S2. Characteristics of patients (matching 1; cirrhosis, genotype; 2:1; in order of appearance).

	patients with statin use at TS	patients without statin use at TS
**number of patients [n]**	**41**	**82**
age [years; median, range]	63 (31-80)	56 (24-78)
sex [n, %]		
male	23 (56.1)	36 (43.9)
female	18 (43.9)	46 (56.1)
genotype [n, %]		
1	30 (73.2)	60 (73.2)
1/2	0 (0)	0 (0)
1/4	0 (0)	0 (0)
1/6	1 (2.4)	2 (2.4)
2	7 (17.1)	14 (17.1)
2/4	0 (0)	0 (0)
3	1 (2.4)	2 (2.4)
4	0 (0)	0 (0)
5	1 (2.4)	2 (2.4)
6	0 (0)	0 (0)
n/a	1 (2.4)	2 (2.4)
used HCV regimen [n, %]		
ELB/GRZ	15 (36.6)	0 (0)
SOF/LDV±RBV	10 (24.4)	25 (30.5)
SOF/RBV±PEG-IFN	4 (9.8)	35 (42.7)
GLE/PIB	3 (7.3)	3 (3.6)
VEL/SOF±RBV	3 (7.3)	5 (6.1)
OMB/PTV/r/DSV±RBV	3 (7.3)	5 (6.1)
OMB/PTV/r±RBV	0 (0)	0 (0)
SOF/DAC±RBV	1 (2.4)	4 (4.9)
SOF/SIM±RBV	1 (2.4)	5 (6.1)
VEL/SOF/VOX	1 (2.4)	0 (0)
SIM/RBV/PEG-IFN	0 (0)	0 (0)
cirrhosis [n, %]	9 (22.0)	18 (22.0)
n/a	0 (0)	0 (0)
statins [n, %]		
simvastatin	16 (39.0)	0 (0)
pravastatin	10 (24.4)	0 (0)
atorvastatin	9 (22.0)	0 (0)
fluvastatin	6 (14.6)	0 (0)

TS therapy start, n/a not applicable, HCV hepatitis C virus, OMB ombitasvir, PTV paritaprevir, r ritonavir, DSV dasabuvir, ELB elbasvir, GRZ grazoprevir, VEL velpatasvir, SOF sofosbuvir, GLE glecaprevir, PIB pibrentasvir, DAC daclatasvir, LDV ledipasvir, RBV ribavirin, SIM simeprevir, VOX voxilaprevir, PEG-IFN pegylated interferon.
