# Supplementary material for: Interdependent Impact of Lipoprotein Receptors and Lipid-Lowering Drugs on HCV Infectivity
Source: Cells. 2021 Jun 29;10(7):1626. doi: 10.3390/cells10071626 (PMC8303410; doi:10.3390/cells10071626)
Supplement: Supplementary file 1 [file cells-10-01626-s001.zip › Table S3.pdf]

Table S3. Characteristics of patients (matching 2; cirrhosis, genotype, regimen; 2:1; in order of appearance).

	patients with statin use at TS	patients without statin use at TS
**number of patients [n]**	**38**	**76**
age [years; median, range]	56 (38-80)	56 (18-77)
sex [n, %]		
male	22 (57.9)	41 (53.9)
female	16 (42.1)	35 (46.1)
genotype [n, %]		
1	30 (79.0)	60 (79.0)
1/2	0 (0)	0 (0)
1/4	0 (0)	0 (0)
1/6	0 (0)	0 (0)
2	6 (15.8)	12 (15.8)
2/4	0 (0)	0 (0)
3	1 (2.6)	2 (2.6)
4	0 (0)	0 (0)
5	1 (2.6)	2 (2.6)
6	0 (0)	0 (0)
n/a	0 (0)	0 (0)
used HCV regimen [n, %]		
ELB/GRZ	15 (39.5)	30 (39.5)
SOF/LDV±RBV	8 (21.1)	16 (21.1)
SOF/RBV±PEG-IFN	4 (10.5)	8 (10.5)
GLE/PIB	3 (7.9)	6 (7.9)
VEL/SOF±RBV	3 (7.9)	6 (7.9)
OMB/PTV/r/DSV±RBV	3 (7.9)	6 (7.9)
OMB/PTV/r±RBV	0 (0)	0 (0)
SOF/DAC±RBV	0 (0)	0 (0)
SOF/SIM±RBV	1 (2.6)	2 (2.6)
VEL/SOF/VOX	1 (2.6)	2 (2.6)
SIM/RBV/PEG-IFN	0 (0)	0 (0)
cirrhosis [n, %]	9 (23.7)	18 (23.7)
n/a	0 (0)	0 (0)
statins [n, %]		
simvastatin	16 (42.1)	0 (0)
pravastatin	7 (18.4)	0 (0)
atorvastatin	9 (23.7)	0 (0)
fluvastatin	6 (15.8)	0 (0)

TS therapy start, n/a not applicable, HCV hepatitis C virus, OMB ombitasvir, PTV paritaprevir, r ritonavir, DSV dasabuvir, ELB elbasvir, GRZ grazoprevir, VEL velpatasvir, SOF sofosbuvir, GLE glecaprevir, PIB pibrentasvir, DAC daclatasvir, LDV ledipasvir, RBV ribavirin, SIM simeprevir, VOX voxilaprevir, PEG-IFN pegylated interferon.
