# Supplementary figures and images for: Interdependent Impact of Lipoprotein Receptors and Lipid-Lowering Drugs on HCV Infectivity
Source: Cells. 2021 Jun 29;10(7):1626. doi: 10.3390/cells10071626 (PMC8303410; doi:10.3390/cells10071626)

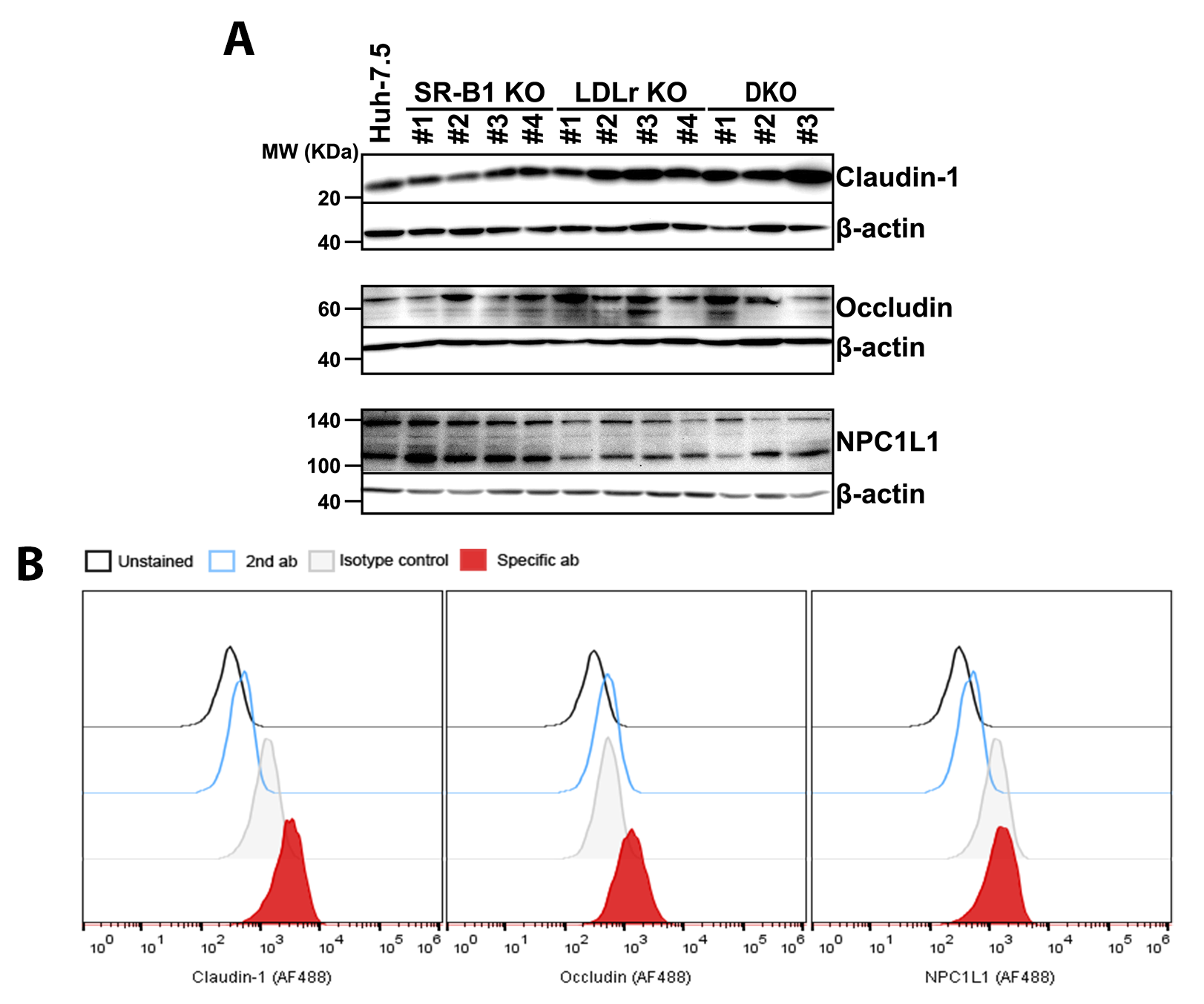

Supplement: Supplementary file 1 [file cells-10-01626-s001.zip › Figure S1.tif]

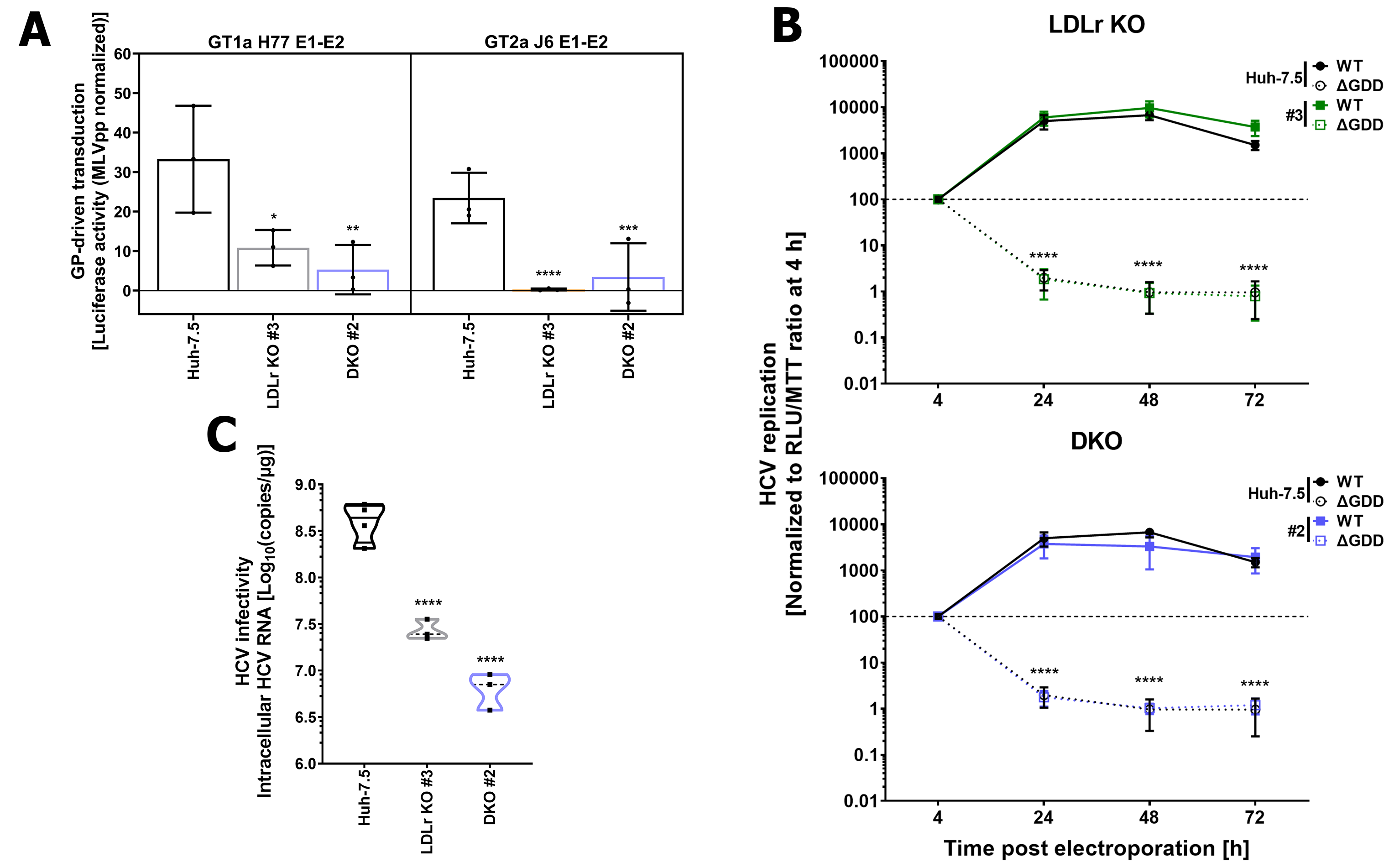

Supplement: Supplementary file 1 [file cells-10-01626-s001.zip › Figure S2.tif]
